# Fecal Components Modulate Human Astrovirus Infectivity in Cells and Reconstituted Intestinal Tissues

**DOI:** 10.1128/mSphere.00568-19

**Published:** 2019-12-18

**Authors:** Francisco J. Pérez-Rodriguez, Gael Vieille, Lara Turin, Soner Yildiz, Caroline Tapparel, Laurent Kaiser

**Affiliations:** a Laboratory of Virology, Division of Laboratory Medicine, Geneva University Hospitals, Geneva, Switzerland; b Department of Microbiology and Molecular Medicine, Faculty of Medicine, University of Geneva, Geneva, Switzerland; c Department of Internal Medicine Specialties, Faculty of Medicine, University of Geneva, Geneva, Switzerland; U.S. Centers for Disease Control and Prevention

**Keywords:** astrovirus, enteric bacteria, viral transmission, virus and bacteria interactions

## Abstract

Human astroviruses (HAstV) are among the most common causative agents of viral gastroenteritis, especially in children, and extraintestinal manifestations have also been described. These viruses are transmitted by the fecal-oral route, implying that stool composition and the gut microbiota may impact their ability to remain infectious. For some enteric viruses, individual bacterial envelope components and other polysaccharide-containing molecules, which are abundant in stools, have been shown to enhance capsid stability. However, the role of the complex stool environment and, most importantly, the role of interindividual differences have been poorly studied. We used HAstV as a model to investigate how the stool environment in itself, its interindividual variability, and some specific stool components could affect HAstV stability and infectivity. Using two different HAstV genotypes, we found that stools as a whole modulate astrovirus infectivity not only in an individual-dependent manner but also in a manner that depends on the viral genotype. A virus-protective effect was observed after incubation with various Gram-positive and Gram-negative bacteria as well as with bacterial components, such as lipopolysaccharide and peptidoglycan. These results were further confirmed in human intestinal tissues, a more physiologically relevant system. Astrovirus infectivity was also preserved by mucin, a major component of intestinal mucus. We further confirmed that these components stabilize the viral capsid. These results show that although HAstV benefits from the stabilizing effect of fecal components, the complexity and variability of the stool composition and the multiple potential interactions may explain the interindividual differences in viral transmission observed in real life.

**IMPORTANCE** To ensure transmission, enteric viruses must maintain their infectivity during the various environmental challenges that they face in transit within and between hosts. Increased knowledge of the factors affecting enteric virus survival may help to control their transmission. This study reveals that specific fecal bacterial components preserve classic human astrovirus infectivity by stabilizing viral particles. However, the outcomes of stool-virus interactions are very variable, ranging from protection to a reduction of viral infectivity, depending on the viral genotype and the individual from whom the stool has been collected. We show that the transmissibility of enteric viruses is dependent on the intestinal contents of the infected individual and highlight the complex multiple interactions that could explain the stochastic nature of enteric virus transmission in humans.

## Abstract (precis)

To ensure transmission, enteric viruses must maintain their infectivity during the various environmental challenges that they face in transit within and between hosts. Increased knowledge of the factors affecting enteric virus survival may help to control their transmission. This study reveals that specific fecal bacterial components preserve classic human astrovirus infectivity by stabilizing viral particles. However, the outcomes of stool-virus interactions are very variable, ranging from protection to a reduction of viral infectivity, depending on the viral genotype and the individual from whom the stool has been collected. We show that the transmissibility of enteric viruses is dependent on the intestinal contents of the infected individual and highlight the complex multiple interactions that could explain the stochastic nature of enteric virus transmission in humans.

## INTRODUCTION

Astroviruses are nonenveloped positive-sense, single-stranded RNA viruses. Currently, four species of astroviruses (*Mamastrovirus 1* [*MAstV 1*], *MAstV 6*, *MAstV 8*, and *MAstV 9*) have been described in humans (1). The members of *MAstV 1* are known as classic human astroviruses (HAstV), while the other three species were discovered more recently and are collectively known as novel human astroviruses. Classic HAstV include eight different genotypes (HAstV-1 to HAstV-8) and are a common cause of acute viral gastroenteritis worldwide, especially in children, although adults can also be affected (1, 2). These viruses can also cause lethal disseminated infections in immunocompromised children (3). HAstV-1 is generally the most frequent genotype detected in stool and wastewater samples (4, 5).

Astroviruses are generally transmitted via the fecal-oral route, as shown in studies with human volunteers (6, 7), through fecally contaminated food, water, or fomites (8). During their transit within and between hosts, enteric viruses must face temperature and pH changes, dehydration, the action of digestive enzymes, and other environmental insults. Successful transmission of an enteric virus relies on the shedding of high loads of viral progeny and the maintenance of persistence (referred to here as the capacity of a virus to retain its infectivity in a given scenario [9]). Persistence depends not only on the type of virus but also on many physical, chemical, and biological factors. Although enteric viruses are in close association with the fecal content over successive infections, the effect of the stools on the infectivity of enteric viruses has been poorly studied (10, 11). Enteric viruses encounter many bacteria during their passage through the gastrointestinal tract and within stools. Bacteria, bacterial surface molecules like lipopolysaccharides (LPS) and peptidoglycans (PGN), and some other polysaccharide-containing molecules can improve the stability of certain enteric viruses, e.g., poliovirus and reovirus (12–15). It has been suggested that the interaction with the aforementioned substances prevents premature RNA release from these viruses (16) and may also reduce conformational changes in the viral particles that could impede attachment to host cells (12). Besides their role in virion stabilization, bacterial surface molecules can also increase virus binding to its host receptor (16). Moreover, the effect of bacteria or their surface molecules on viral stability can differ even between phylogenetically related viruses (15). On the other hand, these molecules can also destabilize viruses, as has been demonstrated for influenza A virus, which uses the fecal-oral transmission route in wild birds (61). Besides direct interactions with enteric viruses, other microbial constituents can affect viral infections indirectly, for instance, by regulating the host immune response (17). Bacterial communities can also modulate the expression of cellular receptors, altering the probability that viruses will bind to and infect host cells (18).

In this study, we evaluated the influence of human stool samples, fecal bacteria, and bacterial surface molecules on the infectivity of two different genotypes of classic human astroviruses (HAstV-1 and HAstV-8) that were subjected to a partially inactivating thermal treatment. We show that the effects of the human stool samples are variable and depend on the sample and the viral genotype. We observed that bacteria and bacterial surface molecules attenuate the loss of HAstV infectivity induced by thermal treatment both in Caco-2 cells and in a three-dimensional tissue model of the human small intestine. We further demonstrate that this effect is related to an increase in the thermal stability of HAstV particles.

## RESULTS

### Human stool samples affect astrovirus infectivity in an individual- and genotype-dependent manner.

**FIG 1:**
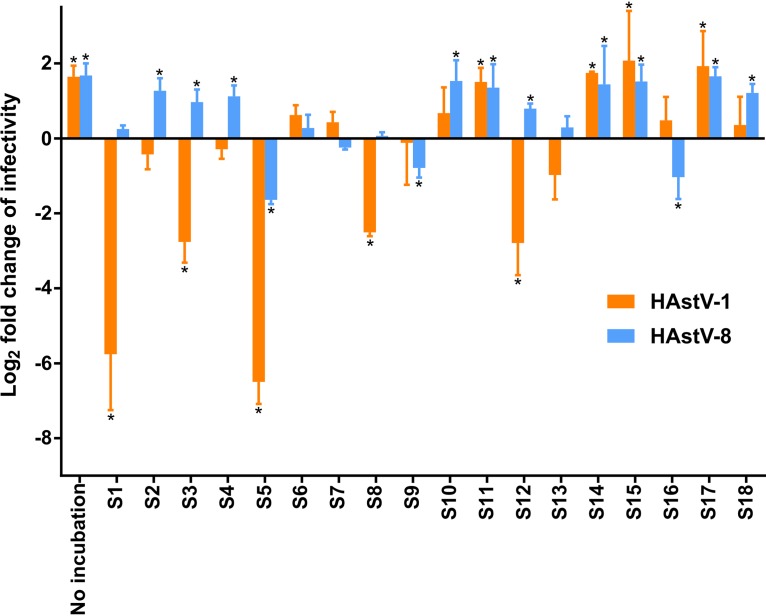
Effect of human stool samples on astrovirus infectivity. HAstV-1 and HAstV-8 were incubated for 4 h at 37°C in the presence of PBS or different filtered human stool samples. Following the incubation, the samples were inoculated into Caco-2 cells (MOI = 0.07), and the infectivity was assessed at 20 hpi by counting the number of infected cells detected by immunofluorescence. The bars represent the log_2_ fold change in infectivity relative to that of viruses incubated with PBS only. Data represent the mean ± standard error of the mean (SEM). *, *P* < 0.05.

We tested the effects of different substances on two genotypes of human astroviruses (HAstV-1 and HAstV-8) that were challenged with a mild thermal treatment. HAstV-1 or HAstV-8 was incubated with phosphate-buffered saline (PBS; used as a control for viral inactivation), filtered human stools from healthy volunteers (assessed to be negative for astrovirus, norovirus genogroups I and II, sapovirus, rotavirus, adenovirus, and enterovirus by reverse transcription [RT]-quantitative PCR [qPCR]), IgA eluates, heat-inactivated bacteria, bacterial surface molecules, or other saccharide-containing molecules for 4 h at 37°C. The samples were inoculated into Caco-2 cells, and virus infectivity was estimated by counting the number of infected cells detected by immunofluorescence at 20 h postinoculation (hpi). Both HAstV-1 and HAstV-8 were sensitive to this mild thermal inactivation, with an average of a 76% loss of infectivity for HAstV-1 and a 70% loss of infectivity for HAstV-8 being found after 4 h of incubation at 37°C in PBS. The effect of the 18 filtered human stool samples tested (samples S1 to S18) was variable, showing in most cases either infectivity enhancement or infectivity inhibition compared to the effect of incubation only with PBS on the viruses (Fig. 1). Globally, 10 (55.6%) of the tested stool samples improved the virus infectivity of HAstV-8, and only 4 of those were also protective for HAstV-1. Nine (50%) and five (27.8%) samples did not significantly affect the infectivity of HAstV-1 and HAstV-8, respectively. An opposite finding was that five (27.8%) and three (16.7%) stool samples significantly reduced HAstV-1 and HAstV-8 infectivity, respectively. Only one of these samples inhibited both genotypes, while the rest of the samples acted in a genotype-specific manner. The possibility of an effect due to cell toxicity could be excluded via a water-soluble tetrazolium salt 1 (WST-1) assay (data not shown). IgA antibodies from samples S5 and S12 were purified and incubated with HAstV-1 and HAstV-8 in order to test for the presence of neutralizing antibodies. IgA eluates from stool sample S5 did not significantly affect either HAstV-1 or HAstV-8 (see Fig. S1A in the supplemental material). In contrast, eluates from stool sample S12 significantly reduced the infectivity for both genotypes (Fig. S1B). Interestingly, the nature of the inhibitory substances seemed to be affected by long-term storage, as some stool samples (stool samples S1, S3, S8, and S16) lost their inhibitory effect after 1 year of storage at −80°C.

10.1128/mSphere.00568-19.1 FIG S1 Effect of IgA purified from human stool samples on HAstV. HAstV-1 or HAstV-8 was incubated for 4 h at 37°C in the presence of filtered stool sample (S) S5 (panel A) or S12 (panel B) or IgA eluates (E) from peptide M columns loaded with sample S5 (panel A) or S12 (panel B). Following the incubation, the samples were inoculated into Caco-2 cells (MOI = 0.07), and the infectivity was assessed at 20 hpi by counting the number of infected cells detected by immunofluorescence. The bars represent the log_2_ fold change in infectivity relative to that for viruses incubated with PBS only. Data represent the mean ± standard error of the mean (SEM). *, *P* < 0.05. Download FIG S1, PDF file, 0.03 MB. Copyright © 2019 Pérez-Rodriguez et al. 2019 Pérez-Rodriguez et al. This content is distributed under the terms of the Creative Commons Attribution 4.0 International license.

To understand the possible role of the fecal microbiota on HAstV infectivity, the bacterial compositions of the stool samples used in viral infections were evaluated using 16S rRNA gene-specific next-generation sequencing. The most abundant phyla observed were *Firmicutes*, *Bacteroidetes*, and, to a lesser extent, *Actinobacteria* and *Proteobacteria* (Fig. S2). There were no significant changes in the alpha or beta diversity of the samples when grouped in terms of their effect on HAstV-1 or HAstV-8 infectivity (Fig. S3A to D). Although no bacterial taxa showed a positive correlation with the infectivity of either virus, the relative abundance of an operational taxonomic unit (OTU) from the genus *Blautia* (OTU identifier 370183) showed a high negative correlation with the infectivity of HAstV-8 (Pearson correlation coefficient [*r*] = −0.81) (Fig. S4).

10.1128/mSphere.00568-19.2 FIG S2 Bacterial composition of stool samples taken from 18 healthy individuals and 4 negative controls based on the sequencing data for the 16S rRNA gene V4 segment. The relative abundance of each phylum is represented as a percentage. Download FIG S2, PDF file, 0.02 MB. Copyright © 2019 Pérez-Rodriguez et al. 2019 Pérez-Rodriguez et al. This content is distributed under the terms of the Creative Commons Attribution 4.0 International license.

10.1128/mSphere.00568-19.3 FIG S3 Alpha and beta diversity of stool samples. (A and B) Alpha diversity, measured by use of the Shannon diversity index, is plotted for samples with a protective effect, an inhibitory effect, or no effect on HAstV-1 (A) and HAstV-8 (B) infectivity. (C and D) Two-dimensional principal-component analysis plots of beta diversity analysis using weighted UniFrac distance matrices. The effect of the samples on HAstV-1 (C) or HAstV-8 (D) infectivity is depicted by different colors: blue, inhibitory effect; green, protective effect; orange, no effect. Download FIG S3, PDF file, 0.04 MB. Copyright © 2019 Pérez-Rodriguez et al. 2019 Pérez-Rodriguez et al. This content is distributed under the terms of the Creative Commons Attribution 4.0 International license.

10.1128/mSphere.00568-19.4 FIG S4 Scatter plot showing the correlation between the abundance of *Blautia* (OTU identifier 370183) in 18 stool samples from healthy individuals and the effect of the samples on HAstV-8 infectivity. Download FIG S4, PDF file, 0.02 MB. Copyright © 2019 Pérez-Rodriguez et al. 2019 Pérez-Rodriguez et al. This content is distributed under the terms of the Creative Commons Attribution 4.0 International license.

### Bacteria and bacterial surface molecules contribute to the preservation of astrovirus infectivity in cell lines and intestinal tissues.

**FIG 2:**
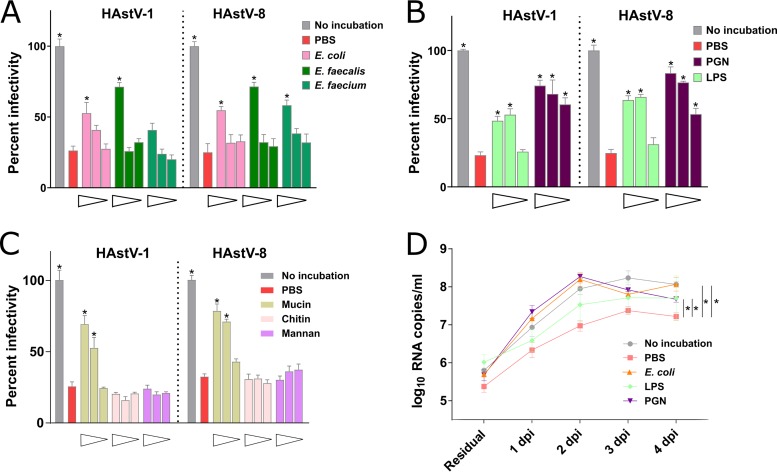
Bacteria, bacterial surface molecules, and mucin reduce astrovirus losses of infectivity after partial thermal inactivation. HAstV-1 and HAstV-8 were incubated for 4 h at 37°C in the presence of PBS or heat-inactivated bacteria (E. coli, E. faecalis, or E. faecium) (A), different bacterial compounds (PGN or LPS) (B), or other polysaccharide-containing molecules (mucin, chitin, or mannan) (C). Decreasing concentrations of bacteria (2.5 × 10^8^, 2.5 × 10^7^, and 2.5 × 10^6^ CFU/ml) and compounds (100 μg/ml, 50 μg/ml, and 10 μg/ml) were tested. Following the incubation, the samples were inoculated into Caco-2 cells (MOI = 0.07), and the infectivity was assessed at 20 hpi by counting the number of infected cells detected by immunofluorescence. The values for infected cells were normalized to those for cells inoculated with control viruses (no incubation at 37°C). (D) Small intestinal tissues were inoculated with HAstV-8 that had not been incubated or that had been incubated for 4 h at 37°C in the presence of PBS, E. coli (2.5 × 10^8^ CFU/ml), LPS (100 μg/ml), or PGN (100 μg/ml). The tissues were inoculated apically and washed 3 times at 4 hpi. Apical samples were collected at the indicated time points, and the extracted viral RNA was quantified. Residual, residual bound virus after 3 washes; dpi, day postinoculation. For panels A to D, data represent the mean ± SEM, and statistical significance relative to the results for virus incubated with PBS only is shown. *, *P* < 0.05.

To further investigate the effect of fecal bacterial communities on HAstV infectivity, we tested three bacterial species isolated from human stools. The incubation of both astrovirus types with heat-inactivated Gram-negative bacteria (Escherichia coli) or Gram-positive bacteria (Enterococcus faecalis and Enterococcus faecium) generally preserved viral infectivity when the bacteria were present at high concentrations (2.5 × 10^8^ CFU/ml) (Fig. 2A). No significant differences were found when HAstV-1 was incubated with Enterococcus faecium (Fig. 2A). We next tested two important components of the bacterial surface related to infectivity maintenance, i.e., lipopolysaccharide (LPS; from Escherichia coli O111:B4) and peptidoglycan (PGN; from Bacillus subtilis). The incubation with LPS or PGN also preserved viral infectivity compared to that of PBS-incubated viruses (Fig. 2B). The effect was similar for both genotypes and showed a dosage dependence (>50 μg/ml for LPS and >10 μg/ml for PGN). These data indicate that the presence of bacteria or widely expressed bacterial surface molecules, such as LPS and PGN, enhances HAstV persistence in the presence of mild thermal stress.

We then assessed the effect of nonbacterial components also present in stools. As *N*-acetylglucosamine (GlcNAc)-containing polysaccharides were shown to increase poliovirus thermostability (13), we incubated HAstV-1 and HAstV-8 with three different concentrations of mucin (a GlcNAc-containing glycoprotein and a major component of mucus), chitin (a linear polymer of GlcNAc), and mannan (a GlcNAc-free polysaccharide). Among the three compounds tested, only mucin restored viral infectivity in a concentration-dependent manner (>50 μg/ml; Fig. 2C). Of note, the activity was similar for both HAstV genotypes. The lack of activity for chitin or mannan suggests that HAstV thermostability is independent of the presence of GlcNAc-containing polysaccharides and that another component of mucin is likely involved. Of note, neither the bacteria nor the substances assayed were toxic for the cells at the concentrations tested, as determined by the WST-1 assay (data not shown).

We then used a three-dimensional model of human small intestinal tissue (EpiIntestinal; MatTek Corporation) to confirm the effect of the bacteria and bacterial surface molecules on astrovirus thermal stability in a more physiological system. HAstV-8 was incubated for 4 h at 37°C in the presence of PBS, heat-inactivated Escherichia coli (2.5 × 10^8^ CFU/ml), LPS (100 μg/ml), or PGN (100 μg/ml). The tissues were apically infected, and after extensive washing at 4 hpi, apically released viral RNA was measured daily by RT-qPCR. As reported for other viruses, residual RNA was detected at 4 hpi, despite extensive washes (19). HAstV-8 replication peaked between days 2 and 4 postinfection (Fig. 2D), with a 100-fold increase in the amount of viral RNA compared to the residual amount being detected for all the samples. The level of viral replication was significantly higher from day 1 to day 4 postinfection when the virus was not incubated or was incubated in the presence of E. coli, LPS, or PGN compared to that when it was incubated with PBS (Fig. 2D). It is important to note that HAstV replicates highly efficiently in a human gut tissue model. The results confirmed our *in vitro* findings that bacteria and bacterial surface molecules prevent the loss of astrovirus infectivity observed after partial thermal inactivation.

### Bacterial surface molecules directly enhance the stability of human astrovirus capsid without changing viral attachment to the host cell.

Various mechanisms have been proposed to explain the effects of bacteria and bacterial surface molecules on the environmental stability of some enteric viruses. The effects can be direct or cell mediated. Therefore, we investigated the underlying mechanisms of action in the case of astroviruses. We first evaluated a possible direct action of bacteria and bacterial surface molecules on the viral particles. We tested whether the effect of these substances on HAstV infectivity occurs during the thermal inactivation process or in a subsequent step. PBS, LPS, PGN, and heat-inactivated E. coli or E. faecalis were mixed with HAstV-1 or HAstV-8 before or after incubation of the viruses for 4 h at 37°C. The infectivity of the viruses was assessed by inoculating the samples into Caco-2 cells and quantifying the number of infected cells by immunofluorescence. In contrast to the results observed in the coincubation experiments (Fig. 2A and B), mixing heat-inactivated bacteria or bacterial surface molecules after the incubation did not change viral infectivity compared to that for the control incubated with PBS (Fig. S5A and B). These data indicate that the protection occurs during the stress of thermal inactivation.

10.1128/mSphere.00568-19.5 FIG S5 Bacteria and bacterial surface molecules protect HAstV infectivity during the thermal inactivation process. LPS (100 μg/ml), PGN (100 μg/ml), or heat-inactivated E. coli (2.5 × 10^8^ CFU/ml) or E. faecalis (2.5 × 10^8^ CFU/ml) was added to HAstV-1 (A) or HAstV-8 (B) before or after incubating the viruses for 4 h at 37°C. Nonincubated and PBS-incubated viruses were included as a reference and a control for viral inactivation, respectively. Viral infectivity was then assessed as described in the legend to Fig. 2. Data represent the mean ± SEM. *, *P* < 0.05. Download FIG S5, PDF file, 0.1 MB. Copyright © 2019 Pérez-Rodriguez et al. 2019 Pérez-Rodriguez et al. This content is distributed under the terms of the Creative Commons Attribution 4.0 International license.

**FIG 3:**
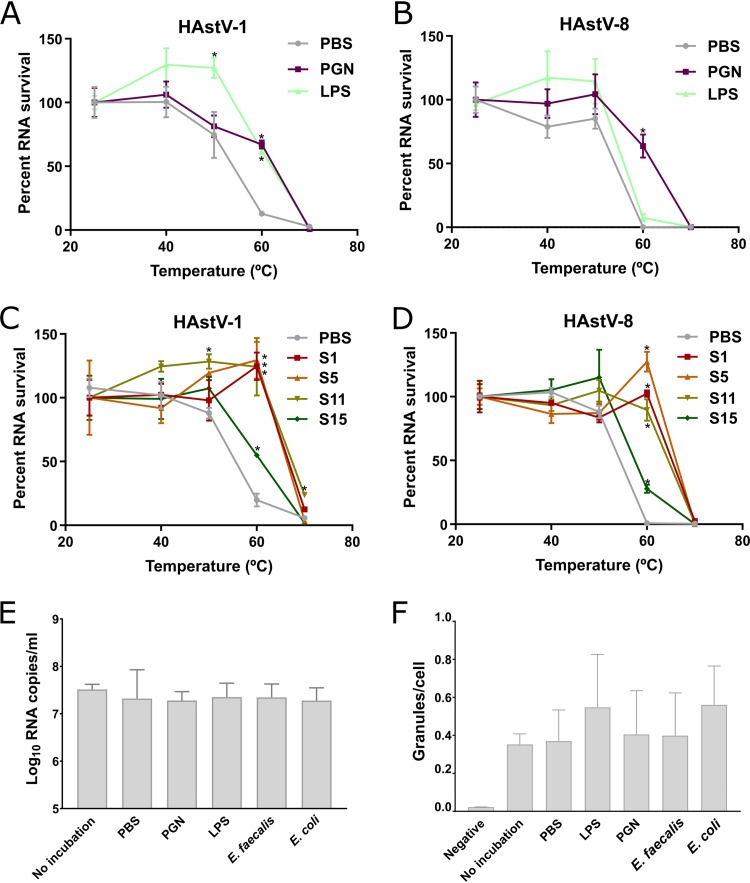
Bacterial surface molecules do not affect viral binding but enhance astrovirus capsid stability. (A to D) RNA exposure assays. Following a preincubation with PBS, LPS (1 mg/ml), or PGN (1 mg/ml) (A, B) or with different stool samples (C, D), HAstV-1 (A, C) and HAstV-8 (B, D) were heated for 2 min at 25°C, 40°C, 50°C, 60°C, or 70°C. The viruses were then treated with RNase for 15 min at 37°C. Intact viral RNA (protected by viral capsids) was extracted and quantified by RT-qPCR. The percentage of RNA copy numbers surviving was calculated by comparison with the starting copy number. (E, F) Binding assays. HAstV-8 was not incubated or incubated for 4 h at 37°C in the presence of PBS, LPS (1 mg/ml), PGN (1 mg/ml), or heat-inactivated E. coli (2.5 × 10^8^ CFU/ml). Viruses were added to Caco-2 cells (MOI = 1 [E] or 2 [F]) for 30 min at 4°C to prevent entry. After extensive washes, the cells were lysed and viral RNA was extracted and quantified (E) or the cells were fixed, astroviruses were immunostained, and the ratio between viral particles (granules) and cells was calculated using a confocal microscope (F). Error bars represent ±SEM. *, *P* < 0.05.

To better define this protective effect and to find out if stool samples confer similar protection, we examined the effects of bacterial surface molecules (LPS and PGN) and four different stool samples (samples S1, S5, S11, and S15) on astrovirus capsid stability. After a preincubation step with or without the aforementioned substances, HAstV-1 and HAstV-8 were heated at various temperatures for 2 min. The samples were then treated with RNase to degrade the exposed RNA in the context of altered capsid structures. The intact capsid-associated RNA was then quantified by RT-qPCR. For both HAstV genotypes, the main differences between treatments were found after incubation at 60°C. At this temperature, the percentages of protected RNA when the viruses were incubated without any substance were 12 to 20% for HAstV-1 and 0.7 to 0.8% for HAstV-8 (Fig. 3A to D). Significantly higher percentages of protected RNA were found when HAstV-1 was incubated with PGN (67.1%) or LPS (62.1%) and when HAstV-8 was incubated with PGN (66.3%) (Fig. 3A and B). In a similar way, all stool samples tested significantly increased the percentage of capsid-associated RNA for both genotypes after incubation at 60°C (Fig. 3C and D). These results confirm that both bacterial surface molecules and stools stabilize human astrovirus particles, protecting the encapsidated genome from external stress.

To exclude the possibility of an additional effect on the virus, we also determined if the mild thermal insult and the presence of bacteria or bacterial surface molecules enhance the ability of astroviruses to attach to cells by performing binding assays. HAstV-8 was added to Caco-2 cells after incubating the virus for 4 h at 37°C in the presence of PBS, LPS, PGN, or heat-inactivated E. coli or E. faecalis. After 30 min of incubation at 4°C to prevent virus entry, the cells were extensively washed and the viral RNA was extracted and quantified or cell-bound viral particles were stained by immunofluorescence and quantified with a confocal microscope. No differences in the amount of bound viral RNA were observed between PBS-incubated and nonincubated control viruses (Fig. 3E). Likewise, no differences in viral RNA levels were found when viruses were incubated with PBS or with heat-inactivated bacteria or bacterial surface molecules (Fig. 3E). In line with these findings, no differences in the amount of bound particles per cell were observed by immunofluorescence (Fig. 3F and Fig. S6). These data suggest that the observed reductions in astrovirus infectivity following a mild thermal treatment are not associated with a decrease in cell binding efficiency. They also indicate that bacteria or bacterial surface molecules do not enhance binding.

10.1128/mSphere.00568-19.6 FIG S6 Representative confocal microscope images of Caco-2 cells incubated with or without HAstV-8. The astrovirus capsid protein is in green, and nuclei stained with DAPI are in blue. Bars, 20 μm. Download FIG S6, PDF file, 1.5 MB. Copyright © 2019 Pérez-Rodriguez et al. 2019 Pérez-Rodriguez et al. This content is distributed under the terms of the Creative Commons Attribution 4.0 International license.

Finally, we investigated whether bacteria, bacterial surface molecules, as well as the different stool samples also favor astrovirus replication indirectly through a cell-mediated effect. Caco-2 cells were incubated for 4 h at 37°C with PBS, LPS, PGN, heat-killed E. coli or E. faecalis, or diverse stool samples (samples S1, S5, S8, S11, S15, or S17) before infection with HAstV-1 or HAstV-8. The substance-containing medium was replaced by fresh medium before the infection. No differences in infectivity were observed after the preincubation of Caco-2 cells with bacteria or bacterial surface molecules (Fig. S7A and B). These results indicate that these substances act directly on the virus rather than indirectly through the cells. Unexpectedly, preincubation of the cells with some stool samples increased the infectivity of HAstV-1 and/or HAstV-8 (Fig. S7C and D). This finding suggests that some stool components can also enhance astrovirus infection in a cell-mediated manner.

10.1128/mSphere.00568-19.7 FIG S7 Exposure of cells to some stool samples but not to bacteria and bacterial surface molecules enhances astrovirus infectivity. Caco-2 cells were exposed to growth medium containing PBS or LPS (33 μg/ml), PGN (33 μg/ml), E. coli (8.3 × 10^7^ CFU/ml), or E. faecalis (8.3 × 10^7^ CFU/ml) (A, B) or different stool samples (1:120 dilution) (C, D). Following a 4-h exposure, the medium was removed and the cells were infected (MOI = 0.07) with HAstV-1 (A, C) or HAstV-8 (B, D). The infectivity was assessed at 20 hpi by counting the number of infected cells detected by immunofluorescence. The values for infected cells were normalized to those for PBS-exposed cells. Download FIG S7, PDF file, 0.05 MB. Copyright © 2019 Pérez-Rodriguez et al. 2019 Pérez-Rodriguez et al. This content is distributed under the terms of the Creative Commons Attribution 4.0 International license.

## DISCUSSION

Temperature is one of the most important factors determining virus inactivation in the environment (20). The incidence of infection with astroviruses is lower during the warmer periods in temperate regions (21–23), possibly due to their thermosensitivity. In our experiments, HAstV incubation at 37°C significantly altered viral infectivity in human intestinal cells and tissues.

In previous studies, feces were considered a simple factor that either enhanced enteric virus survival on food and fomites (10, 24) or produced opposite effects, depending on the temperature and the surface assayed (11). In the present work, we took into account the intersubject variability and complexity of the intestinal contents by using human stool samples from a range of volunteers. We demonstrated that coincubation of astrovirus and stool specimens can affect viral infectivity in various ways, ranging from protection to inhibition, and also in a genotype-dependent manner. Regarding the inhibition found in some cases, our data suggest that it is caused by the presence of IgA in some but not all the cases. Studies on mucosal immunity to astrovirus infections are lacking, but our data suggest that some healthy adults may secrete neutralizing astrovirus-specific IgA, which could conceivably play a role in mediating protective immunity. Studies on rotavirus immunity show that rotavirus-specific gut IgA can persist for several months in both animals and humans (25–27). The inhibition of both genotypes by sample S12 IgA eluates might have been due to the absence in the eluates of substances that protect or increase the infectivity of HAstV-8. On the other hand, the lack of significant inhibitory activity of the IgA eluates from sample S5 suggests that other molecules with anti-HAstV activity could have been present in the sample. Stools can contain a myriad of molecules with potential antiviral activity: innate immune humoral factors (such as lysozyme, interferons, and lactoferrin), dietary compounds (e.g., polyphenols), and microbial compounds (e.g., bacteriocins) (28, 29). An inverse correlation between the relative abundance of a bacterial taxon (an OTU from *Blautia*) in a sample and the effect of that sample on HAstV-8 infectivity was observed. The possibilities of the production of antiviral substances by *Blautia* or a relationship between the mucosal immune system and these bacteria (30), which is associated with reduced death from graft-versus-host disease (31), deserve to be explored.

We found that different heat-inactivated bacteria and broadly expressed bacterial envelope components were generally protective for astrovirus infectivity in the presence of thermal treatment. These observations were confirmed using a three-dimensional tissue model of the human small intestine. This is the first time, to the best of our knowledge, that HAstV has been grown in intestinal tissues. The use of human tissues, which retain the cellular diversity and organization present in the small intestine (32), may help to advance the study of HAstV biology, as infections in these tissue models are more representative of infections *in vivo*.

While a density of 10^11^ bacteria per gram of stool has been reported in different studies (33), acutely infected individuals can excrete from 10^5^ to 10^11^ viral particles per gram of stool (34). Given that, the bacterium-to-astrovirus ratio shown to enhance thermostability in our experiments (∼2 × 10^4^:1) can occur in natural infections. The bacterial polysaccharide concentration shed in the gastrointestinal tract is likely to be on the order of 10s of micrograms per milliliter (35). This predicted concentration is also within the range of concentrations that our results show to be protective for HAstV infectivity (from 50 μg/ml for LPS and from 10 μg/ml for PGN). In another study, similar concentrations of LPS and PGN were needed to protect reovirus infectivity (12).

Other polysaccharide-containing molecules of nonbacterial origin can also affect the stability of some enteric viruses. It has been shown that only *N*-acetylglucosamine-containing polysaccharides stabilize poliovirus (13). In this study, we found that chitin, a polymer of *N*-acetylglucosamine, does not protect HAstV against thermal inactivation. This supports the reported observation that stabilization by polysaccharides or glycoproteins depends on the characteristic of the enteric virus (12). In addition, we observed that mucin protects astrovirus infectivity at concentrations similar to those at which LPS is protective. This is unlike what was shown for poliovirus, where LPS was reported to be protective from concentrations 20 times lower than the concentration of mucin that was protective (13). High concentrations of bacterial surface molecules and/or mucin (and possibly some dietary compounds that were not tested), combined with the absence of inhibitors, may explain the protective effect of many stool specimens on viral infectivity in the coincubation experiments, while the ubiquitous expression of peptidoglycan across bacteria, as well as the additive effect of host or dietary molecules, such as mucin, may explain the absence of positive correlations with any specific taxa in the microbiota analysis.

There was no impact of bacteria or bacterial surface molecules on astrovirus infectivity when they were added after the thermal treatment. This suggests that the main benefit of these substances is the protection of the particles against the inactivation caused by heat. This hypothesis is supported by the RNase RT-qPCR experiments. Both viruses tested (HAstV-1 and HAstV-8) were, in general, more stable in the presence of bacterial surface molecules, suggesting that stool components can modulate viral infectivity by preserving intact viral particles. These components may permit the survival of unstable virions that might otherwise prematurely eject their genetic content. An increased stability of the viral particles was also observed when different stool specimens were tested (including some that were inhibitory in the coincubation infectivity experiments), similar to the findings described for feline calicivirus (36).

Bacterial surface glycans can also benefit viruses through other direct interactions. These molecules can stabilize viral particles but also enhance binding to viral receptors (12–14, 16). Our binding assays suggested that HAstV attachment to host cells is not altered by incubation at 37°C. Thus, viral particles may have lost their infectivity without losing their cellular affinity. Moreover, in contrast to poliovirus (16), bacterial polysaccharides did not enhance astrovirus attachment to the cell surface in our setting.

Microbiota can modulate the outcome of viral infections not only through a direct interaction but also indirectly in a cell-dependent manner. We could not demonstrate an indirect effect of bacteria, LPS, or PGN on astrovirus infectivity. An unexpected finding was that the preincubation of cells with stools (including samples that had an inhibitory effect when coincubated with HAstV-1 or -8) increased susceptibility to viral infection.

In this study, we investigated how human stools and various stool components can influence the persistence of a human virus that causes gastroenteritis when faced with thermal stress. We demonstrated that human stools can affect astrovirus in various ways: protecting the viral particles against thermal inactivation, inhibiting the infectivity in some instances, or increasing the infectivity in an indirect way. As we showed, bacterial surface molecules (and probably also other polysaccharide-containing molecules) are responsible for the first effect, IgA partially explains the second effect, while further experiments are pending to elucidate the molecules responsible for the last effect. Stools are composed of water, ash, bacterial biomass, polysaccharides, proteins, fat, and other undigested food residues (37). Fecal composition is highly variable, with diet being the major cause of the variation (37, 38). Thus, bacterial mass accounts for between 30% and 60% of the fecal dry weight in healthy subjects (37), and LPS levels in human feces can range from 0.01 to 100 mg per gram of stool (39). Moreover, the composition of the gut microbiota is different in every person and depends on host genetics and environmental factors (early microbial exposure, diet, antibiotics, etc.) (40–43). Quantitative and qualitative differences in the various active stool components among individuals might explain the variable outcomes observed in Fig. 1. More generally, our investigation highlights that the specific stool composition impacts the stability and infectivity of enteric viruses.

## MATERIALS AND METHODS

### Cells, tissues, and viruses.

Cells of the colon adenocarcinoma cell line Caco-2 were grown in minimum essential medium supplemented with GlutaMAX (MEM; Gibco, Thermo Fisher Scientific), nonessential amino acids, antibiotics (penicillin and streptomycin), and 10% fetal bovine serum (FBS). Small intestinal tissues (EpiIntestinal) were obtained from MatTek (Ashland, MA, USA) and maintained by adding on a daily basis 200 μl apical and 1.5 ml basolateral SMI-100-MM medium. Cells and tissues were kept at 37°C in a 5% CO_2_ atmosphere.

A human astrovirus belonging to genotype 8 (HAstV-8) detected in a recent study carried out in Geneva, Switzerland (44), was propagated in Caco-2 cells. For isolation of the virus, a stool sample was diluted 1:10 in PBS and filtered through a 0.45-μm-pore-size membrane. After a 30-min incubation with trypsin (10 μg/ml), this material was inoculated into Caco-2 cells. An hour later, the inoculum was removed, the cells were washed, and minimum essential medium with 5 μg/ml trypsin was added. After incubating the cells at 37°C for 3 days, the medium was centrifuged for 10 min at 1,500 × *g* and the virus-containing supernatant was collected. The virus was serially passaged in Caco-2 cells using the same procedure. The presence and identity of the virus were confirmed by RT-qPCR, immunofluorescence using monoclonal antibody 8E7 (Invitrogen, USA), transmission electron microscopy, and capsid sequencing. HAstV-1 was produced from the plasmid pAVIC, which contains the full-length genome of the Oxford strain (45), and was generously provided by A. Bosch and S. Guix (University of Barcelona, Barcelona, Spain). Infectious particles were obtained from pAVIC as previously specified (46), and viruses were serially passaged as specified above for HAstV-8. HAstV-1 and HAstV-8 were concentrated by ultracentrifugation, aliquoted, and stored at −80°C. Viruses from passages 5 to 9 were used. The concentration of infectious particles in every stock was determined by infecting Caco-2 cells with different stock dilutions and counting the numbers of infected cells detected by immunofluorescence.

### Stools, bacteria, and chemicals.

We recruited 18 unrelated healthy volunteers from 21 to 45 years old. The participants had not taken antibiotics in the past month. The subjects were instructed to collect one fecal sample in sterile containers (Sarstedt). Each sample was diluted 1:20 in PBS, aliquoted, and frozen at −80°C upon arrival at the laboratory. Aliquots were centrifuged for 3 min at 100 × *g*, filtered through a 0.45-μm-pore-size membrane before the experiments, and identified as samples S1 to S18.

Two human stool samples were plated in chocolate Polyvitex agar (bioMérieux) under aerobic conditions. After incubation overnight at 37°C, some colonies were identified by matrix-assisted laser desorption ionization–time of flight mass spectrometry. Individual colonies of Escherichia coli, Enterococcus faecalis, and Enterococcus faecium were grown in Luria-Bertani (LB) broth at 37°C. Bacteria were pelleted and resuspended in PBS to a concentration of 5 × 10^8^ CFU/ml. Resuspended bacteria (0.5 ml) were inactivated by heating at 65°C for 30 min. Bacterial inactivation was confirmed by plating on LB agar. Bacterial concentrations were calculated according to the bacterial culture optical density measured at 550 nm. Confirmations of the number of CFU were done by plating on LB agar and incubating overnight at 37°C.

Mannan from Saccharomyces cerevisiae, mucin from porcine stomach, chitin from shrimp shells, lipopolysaccharides (LPS) from Escherichia coli strain O111:B4, and peptidoglycan (PGN) from Bacillus subtilis purified by phenol extraction were obtained from Sigma.

### Ethics statement.

EpiIntestinal tissues were ordered from MatTek, a biotechnology company. This company biobanks tissues from anonymized samples after ethical approval.

Subjects providing stool samples gave informed consent to participate in this study, and the Research Ethics Committee of Geneva (Geneva, Switzerland) approved this consent procedure (project number 2018-01474).

### Infectivity assays.

Astroviruses were initially treated with 10 μg/ml of trypsin-EDTA (Gibco) for 30 min at 37°C (200 μg/ml of trypsin was used for HAstV-8 for the stool incubation experiments, to ensure the maximum viral activation). HAstV-8 and HAstV-1 (1.5 × 10^4^ infectious particles diluted in PBS) were incubated for 4 h at 37°C in a volume of 150 μl in the presence of PBS, filtered human stool samples, IgA eluates, heat-inactivated bacteria (2.5 × 10^8^, 2.5 × 10^7^, and 2.5 × 10^6^ CFU/ml), LPS, PGN, mucin, mannan, or chitin (the last three substances were used at concentrations of 100 μg/ml, 50 μg/ml, and 10 μg/ml). Virus mixed with PBS but not incubated was used as a reference. To examine if bacteria or bacterial compounds act during the thermal insult, heat-inactivated E. coli or E. faecalis (2.5 × 10^8^ CFU/ml) or LPS or PGN (100 μg/ml) was added before or after incubating HAstV-1 and HAstV-8 (1.5 × 10^4^ infectious particles) for 4 h at 37°C.

The infectivity of HAstV in Caco-2 cells was evaluated by inoculating 33 μl of the virus-containing solutions into confluent nonpolarized cells in 96-well microplates (approximately 4.7 × 10^4^ cells/well, multiplicity of infection [MOI] = 0.07) containing 67 μl growth medium, with immunofluorescence being performed 20 h later and the number of infected cells being counted as described below. The percentage of infected cells was normalized to that for cells inoculated with nonincubated viruses to compare samples across replicates.

HAstV-8 replication in small intestinal tissues was assessed by inoculating apically 100 μl of virus-containing solutions. After 4 h at 37°C, the inoculum was removed and the tissues were washed 3 times with 200 μl PBS. Starting at that moment, daily sampling was performed by applying and collecting 200 μl of medium apically. RNA was extracted using an EZNA total RNA kit I (Omega Bio-Tek) according to the manufacturer’s instructions. Viral RNA was quantified by RT-qPCR as described below.

To test if exposure of cells to bacteria, bacterial surface molecules, or stool samples enhances astrovirus infectivity, Caco-2 cells were exposed for 4 h at 37°C to growth medium containing PBS or heat-inactivated E. coli or E. faecalis (8.3 × 10^7^ CFU/ml), LPS or PGN (33 μg/ml), or filtered human stool samples (1:3 dilution) to mimic the concentrations present in the experiments using virus-containing solutions. After the exposure, the medium was removed, fresh medium was added, the cells were inoculated with HAstV-1 or HAstV-8 (3.3 × 10^3^ infectious particles, MOI = 0.07), and the infectivity was assessed as described above.

### Purification of IgA.

IgA antibodies from stool samples were purified using peptide M-agarose beads (InvivoGen), following the manufacturer’s instructions with minimal changes. In brief, purification columns were prepared by loading 350 μl peptide M-agarose into 900-μl columns (Pierce, Thermo Scientific) and washing with PBS. Filtered human stool samples (350 μl) were added to the columns, followed by incubation at room temperature for 45 min. The columns were washed with PBS, IgA antibodies were eluted using 0.1 M glycine-HCl buffer (pH 2.3), and the eluates were immediately neutralized with 1 M Tris HCl (pH 8.7). The eluted fractions were buffer exchanged into PBS and concentrated to a final volume of 350 μl using Amicon Ultra-15 centrifugal filters (Merck KGaA) with a 100-kDa-molecular-mass cutoff.

### Evaluation of cytotoxicity.

The cytotoxicity of the different substances tested in the infectivity assays for Caco-2 cells was evaluated using the cell proliferation reagent WST-1 (Roche). Fresh medium (MEM) was added to confluent nonpolarized Caco-2 cells growing in a 96-well microplate. The substances to be tested were added to the medium at the same concentrations used in the infectivity assays. After incubating for 20 h (37°C, 5% CO_2_), the WST-1 assays were performed according to the manufacturer’s instructions. The absorbance was measured at 450 and 620 nm in a FilterMax F5 multimode microplate reader (Molecular Devices).

### DNA extraction.

DNA was extracted from the stool samples using a PowerLyzer PowerSoil DNA isolation kit (MoBio, Qiagen, USA) according to the manufacturer’s instructions. The DNA concentrations were measured with a NanoDrop ND 1000 spectrophotometer (Thermo Fisher Scientific, USA) and were normalized to a concentration of 50 ng/μl. For determination of potential environmental and/or cross contamination within samples, four empty sample tubes were processed next to the biological samples.

### Library construction and Illumina sequencing.

For each sample, 16S rRNA DNA libraries were generated following a protocol described previously (47). Briefly, the V4 region of the 16S rRNA gene was targeted for PCR amplification in triplicate using 5Prime Hot master mix (QuantaBio, USA) with a modified universal bacterial 16S primer pair (515F/806R) (48). PCR products for each amplicon were pooled, run on a 2% agarose gel for visual confirmation of the libraries, and quantified with a Quant-iT PicoGreen double-stranded DNA (dsDNA) assay kit (Life Technologies, USA). One hundred nanograms of each amplicon was pooled and cleaned using a QIAquick PCR purification kit (Qiagen, USA). The library was then quantified using a Qubit dsDNA BR assay kit (Invitrogen, USA) and mixed with bacteriophage phiX DNA (10%). Paired-end sequencing (250 bp) was performed on a MiSeq platform (Illumina, USA) in iGE3, Institute of Genetics and Genomics in Geneva, CMU, University of Geneva. Sequencing results were obtained and demultiplexed using the standard method supplied by the MiSeq Illumina platform.

### Bioinformatics pipeline.

Sequence reads were analyzed using the software QIIME following a previously described pipeline (47–49) with slight modifications. Briefly, forward and reverse reads were joined with a minimum 200-bp overlap (fastq-join), trimmed, and formed into a single library with a threshold for sequence quality (a Phred score of 20 or higher) (50). Chimeric reads were detected and filtered using the UCHIME program (51). Operational taxonomic units (OTUs) with a 97% sequence identity to a closed reference sequence (Greengenes database, v13.8) were picked (52–54). Using the resultant OTU table, samples were analyzed with respect to alpha diversity (Shannon), beta diversity (UniFrac weighted), and the relative abundance of different taxonomical bacterial communities (55–59). The Pearson correlation coefficients between the relative abundance of different OTUs and the log_2_ fold change in infectivity for each viral genotype were calculated using Excel software (Microsoft). Graphs were produced using QIIME visual representation or GraphPad Prism (v7) software.

### Virus binding assays.

**(i) Assay to quantify viral RNA.** Caco-2 cells were grown in 96-well microplates until confluence. Cells were washed with cold PBS and prechilled for 10 min after the addition of 200 μl cold PBS. The buffer was removed, and 50 μl of chilled virus-containing solution was added to the cells. Virus solutions consisted of 5 × 10^4^ infectious particles (MOI = 1) of trypsin-activated HAstV-8 not incubated or incubated for 4 h at 37°C in the presence of PBS, LPS (1 mg/ml), PGN (1 mg/ml), or heat-inactivated Escherichia coli (2.5 × 10^8^ CFU/ml) or Enterococcus faecalis (2.5 × 10^8^ CFU/ml). The cells were incubated for 30 min on ice and then washed 3 times with 200 μl cold PBS. The cells were lysed with 200 μl TRK lysis buffer (Omega Bio-Tek), and viral RNA was extracted using the EZNA total RNA kit I (Omega Bio-Tek) according to the manufacturer’s instructions. Extracted RNA was quantified by RT-qPCR as described below. Viral RNA genome copy numbers were normalized to the copy number for the RNase P housekeeping gene.

**(ii) Assay to quantify viral particles.** Caco-2 cells were grown on glass coverslips in 24-well plates until confluence. Cells were prewashed, prechilled, incubated at 4°C with different virus-containing solutions, and washed as described above for the previously described assay, but the volumes of the solutions and the number of viral particles added differed. The cells were washed with 500 μl PBS and inoculated with 200-μl virus solutions containing 4 × 10^5^ infectious particles (MOI = 2). Following the washes, the cells were fixed with paraformaldehyde. Astroviruses were stained with astrovirus-specific antibody and goat anti-mouse A488 and cells were stained with DAPI (4′,6-diamidino-2-phenylindole), as described below. Uninfected cells were included as a negative control. Coverslips were mounted in Dako fluorescent mounting medium (Dako). Immunofluorescent images were acquired with a Zeiss LSM 700 Meta confocal microscope using a Plan-Neofluar 63× (numerical aperture, 1.4) oil objective. The number of viral particles per cell in every image was counted using the granularity module of MetaMorph software (v7.10; Molecular Devices), which detects and counts granules associated with cells. The number of granules per cell in at least eight independent visual fields from two independent wells was counted. No fewer than 1,000 cells were analyzed for every condition. The results were expressed as the average number of granules in each cell.

### RNA exposure assays.

PBS, LPS (1 mg/ml), PGN (1 mg/ml), or stool sample S1, S5, S11, or S15 was mixed with 10^5^ RT-qPCR copies of HAstV-1 and HAstV-8 trypsin-activated viruses in a volume of 100 μl and preincubated at 37°C for 30 min. After this preincubation, the viruses were subjected to heat inactivation and an RNase One RT-qPCR assay was performed as described by Topping et al. (36). Samples contained in PCR tubes were heated for 2 min at 25°C, 40°C, 50°C, 60°C, or 70°C in a thermal cycler. Following the heat inactivation step, RNase treatment was carried out by the addition of 10 μl of 10× reaction buffer and 1 μl RNase One RNase (Promega). Control virus solutions containing reaction buffer but not RNase were included to demonstrate the absence of free viral RNA in the initial material. Control RNA solutions containing reaction buffer and RNase or only reaction buffer were included to check for the performance of the RNase. The solutions were incubated at 37°C for 15 min. Viral RNA was extracted using a QIAamp viral RNA minikit (Qiagen) according to the manufacturer’s instructions. RT-qPCR was performed as described below. The percentage of RNA surviving was calculated by comparison with the starting copy number.

### Real-time RT-PCR quantification.

RT-qPCR was performed with a QuantiTect probe RT-PCR kit (Qiagen) in a StepOne Applied Biosystems thermocycler. The astrovirus genome was quantified using previously described primers and probe (60). For preparing a quantitative reference standard, a fragment covering the target was amplified from HAstV-1. Reverse transcription and PCR were performed with Moloney murine leukemia virus reverse transcriptase and DreamTaq DNA polymerase (Thermo Fisher Scientific), respectively. The PCR product was cloned into a pCR4-TOPO cloning vector (Invitrogen, USA), and the plasmid was transformed into chemically competent E. coli One Shot TOP10 cells (Invitrogen, USA). The plasmid was *in vitro* transcribed using a T7 RiboMAX large-scale RNA production system (Promega), and 10-fold dilution series (from 10^7^ to 10^4^ copies/ml) were used as a standard.

### Immunofluorescence.

Caco-2 cells were fixed and permeabilized with cold methanol-acetone (50:50) for 10 min. After being washed with PBS and PBS–0.1% Triton X-100, the cells were incubated for 1 h at 37°C with a 1:5,000 dilution of the monoclonal antibody 8E7 (Invitrogen, USA) in PBS–1% bovine serum albumin (BSA). Three washings with PBS–0.1% Triton X-100 were performed before incubating for 1 h at 37°C with Alexa Fluor 488-conjugated anti-mouse IgG antibody (Invitrogen, USA) at a 1:3,000 dilution in PBS–DAPI–1% BSA. The cells were additionally washed and visualized using an ImageXpress Micro XL (Molecular Devices) microplate reader and a 10× S Fluor objective. The percentage of infected cells was estimated by counting the number of cells expressing astrovirus structural proteins and the total number of cells (DAPI-positive cells) from four different fields per sample using MetaXpress software (Molecular Devices).

### Statistics.

GraphPad Prism (v7.03) software (GraphPad Software) was used for statistical analysis. All values are expressed as the mean ± standard error of the mean (SEM). Statistical significance (*P* < 0.05) was assessed using an analysis of variance followed by Tukey’s test for multiple comparisons. The experiments were done at least in biological triplicate, and in each experiment, conditions were run in duplicate.

### Data availability.

The full sequence of the gene coding for the capsid precursor protein has been submitted to GenBank (accession number MK882944). 16S rRNA gene next-generation sequencing data were deposited in the NCBI BioProject database under accession number PRJNA594378.
