# Supplementary figures and images for: Fecal Components Modulate Human Astrovirus Infectivity in Cells and Reconstituted Intestinal Tissues
Source: mSphere. 2019 Dec 18;4(6):e00568-19. doi: 10.1128/mSphere.00568-19 (PMC6920511; doi:10.1128/mSphere.00568-19)

**A****Log<sub>2</sub> fold change of infectivity****S5**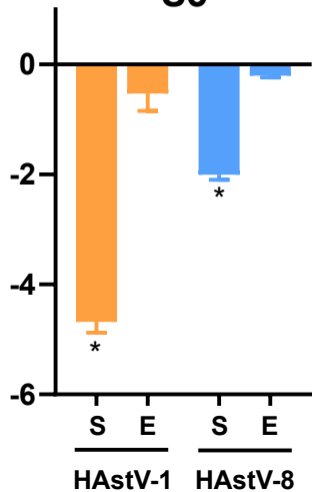**B****Log<sub>2</sub> fold change of infectivity****S12**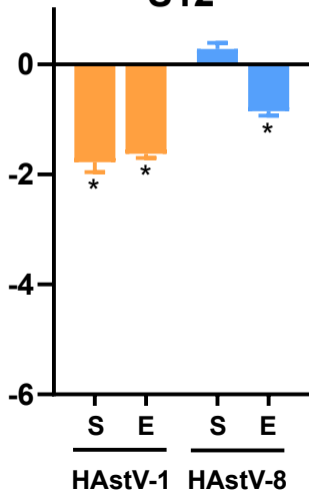

Supplement: FIG S1 [file mSphere.00568-19-sf001.pdf]

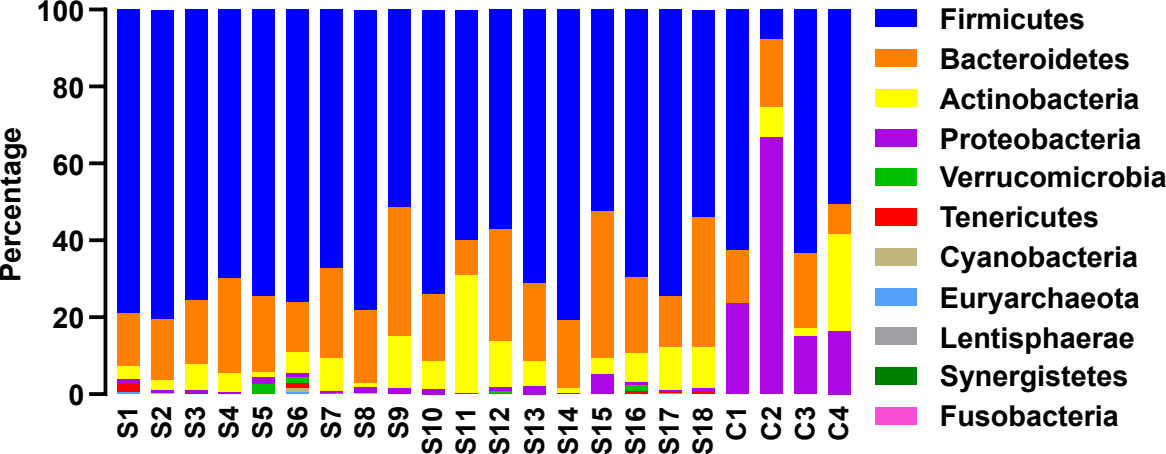

Supplement: FIG S2 [file mSphere.00568-19-sf002.pdf]

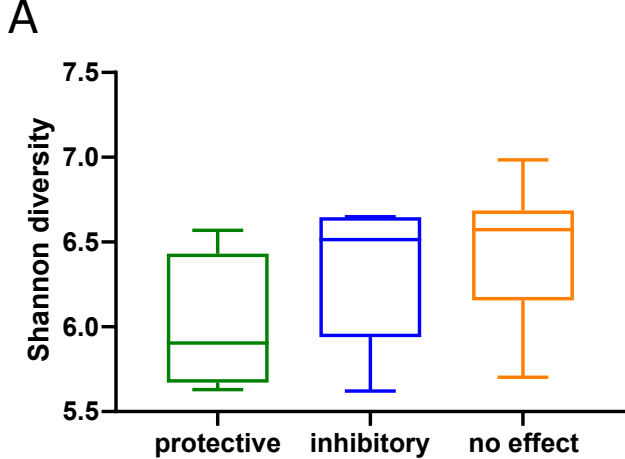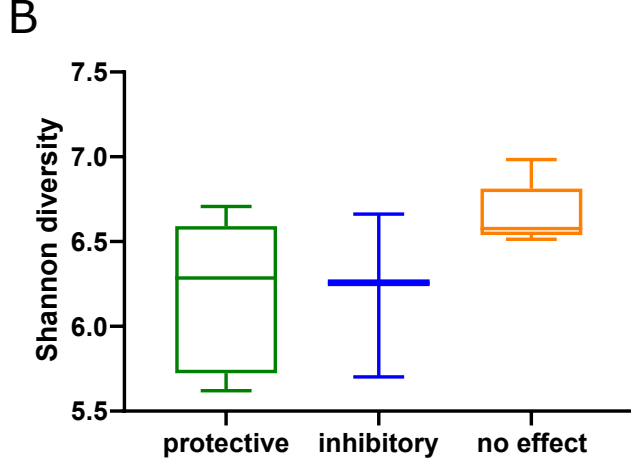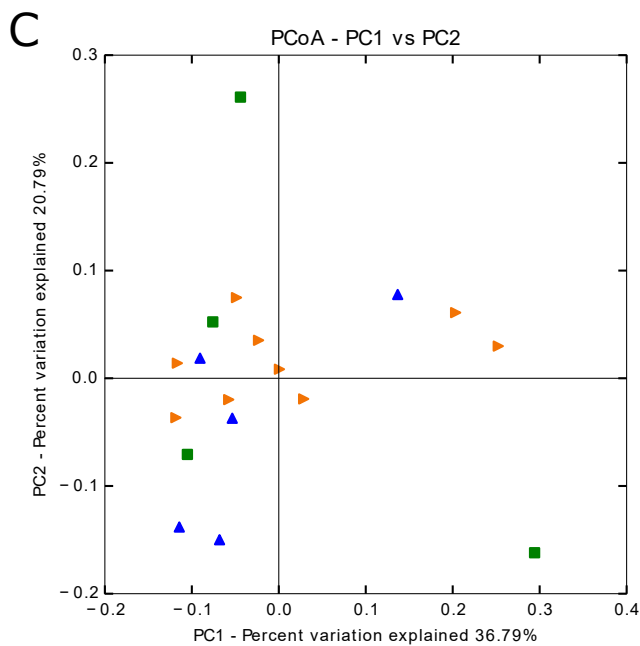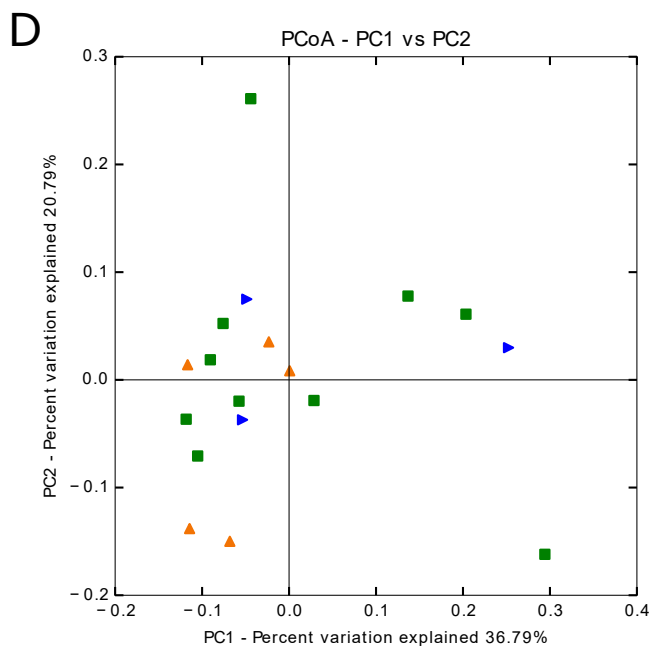

Supplement: FIG S3 [file mSphere.00568-19-sf003.pdf]

***Blautia* (OTU ID 370183)**

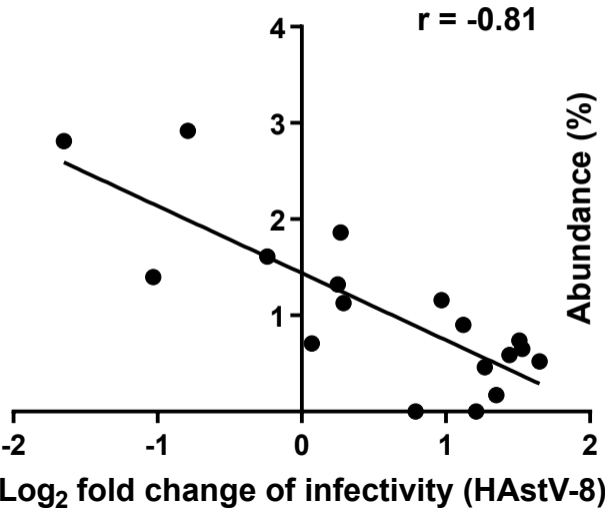

Supplement: FIG S4 [file mSphere.00568-19-sf004.pdf]

**A**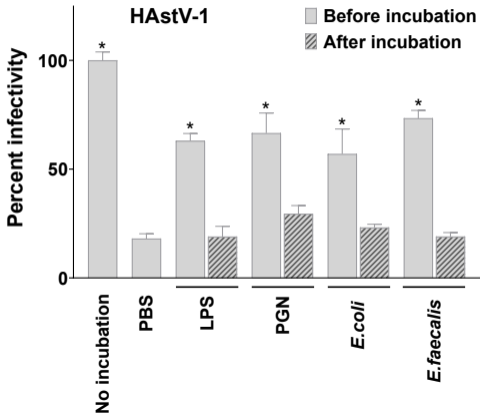**B**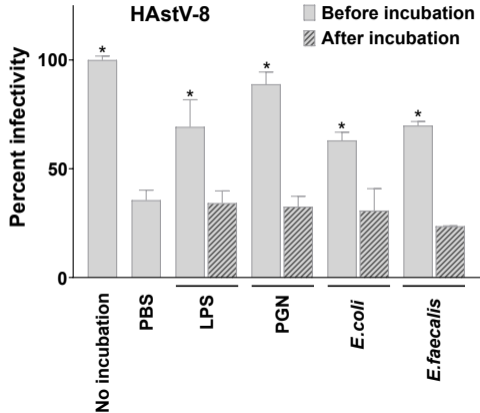

Supplement: FIG S5 [file mSphere.00568-19-sf005.pdf]

**Negative**

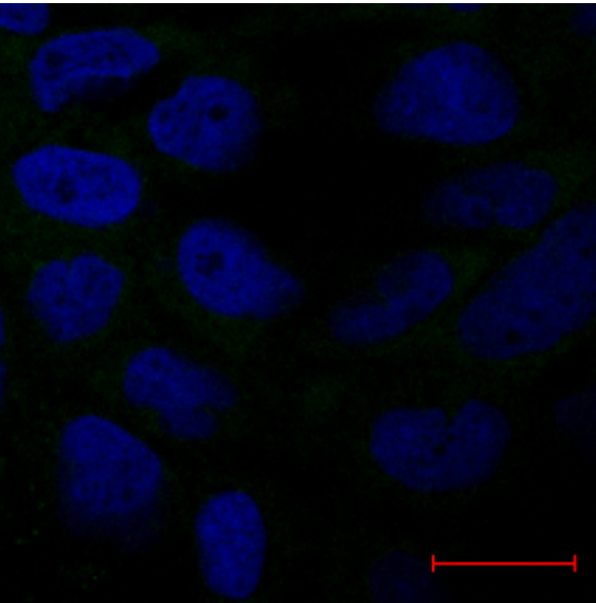

**HAstV-8**

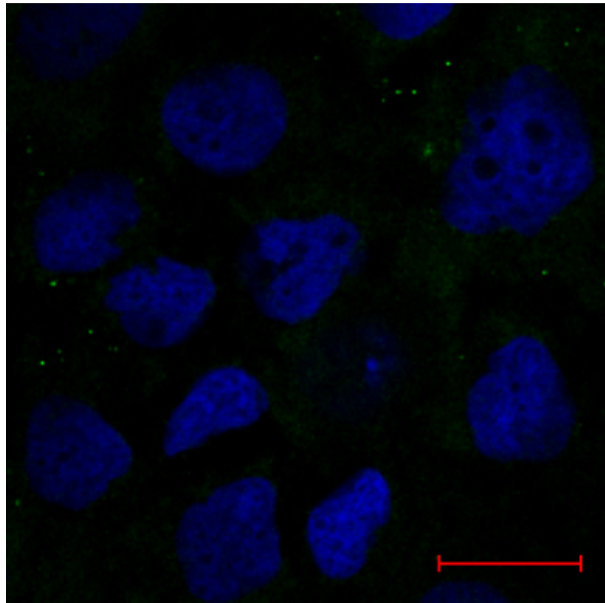

Supplement: FIG S6 [file mSphere.00568-19-sf006.pdf]

**A**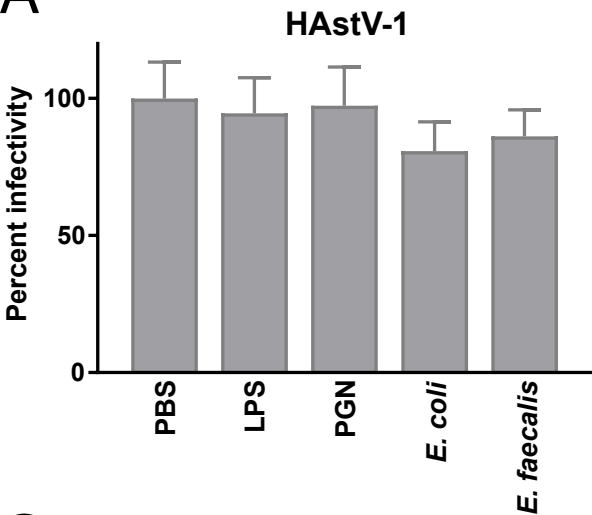**B**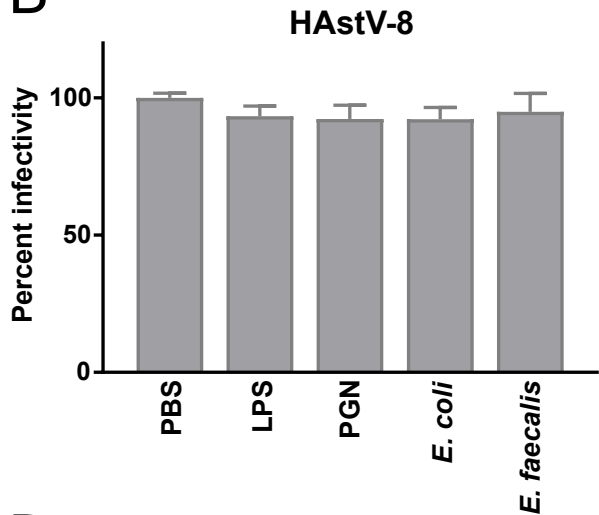**C**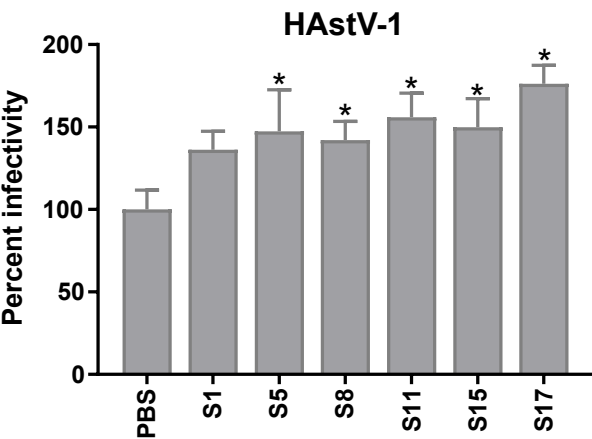**D**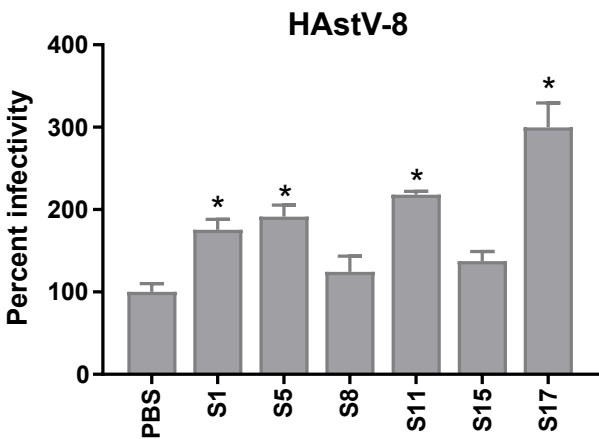

Supplement: FIG S7 [file mSphere.00568-19-sf007.pdf]
